# Supplementary material for: Consumption-based greenhouse gas emissions accounting with capital stock change highlights dynamics of fast-developing countries
Source: Nat Commun. 2018 Sep 4;9:3581. doi: 10.1038/s41467-018-05905-y (PMC6123491; doi:10.1038/s41467-018-05905-y)
Supplement: Supplementary file 1 — Supplementary Information [file 41467_2018_5905_MOESM1_ESM.pdf]

Consumption-based Greenhouse Gas Emissions Accounting with Capital Stock Change Highlights Dynamics of Fast-developing Countries

(Supplementary Information)

Chen *et al.*

Supplementary Figures

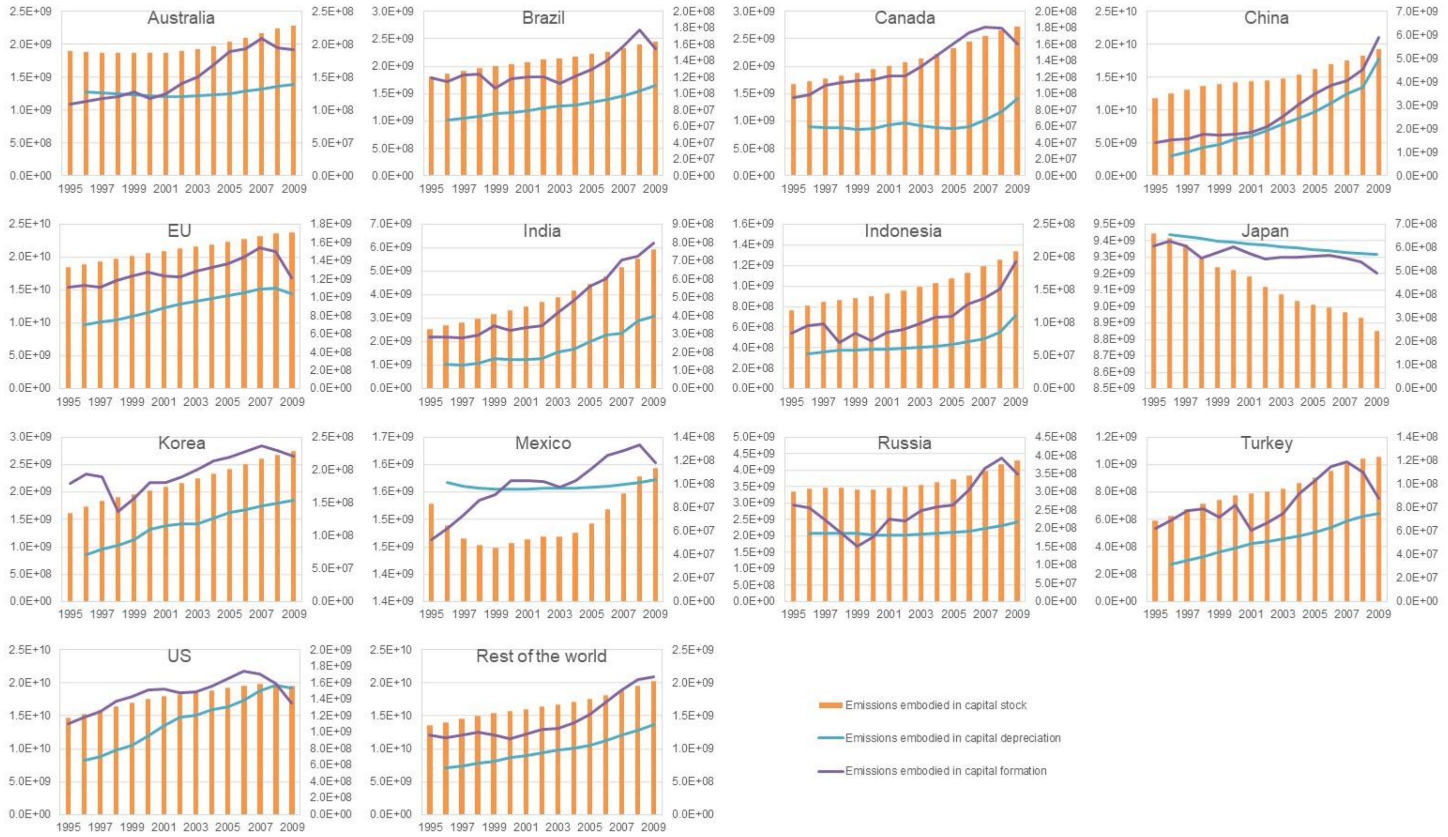

Supplementary Figure 1 Emissions embodied in capital stock, capital depreciation, and capital formation. Left axis indicates value of emissions embodied in capital depreciation and formation and right axis indicates value of emissions embodied in capital stock. Unit of both left and right axes are tCO_2e.

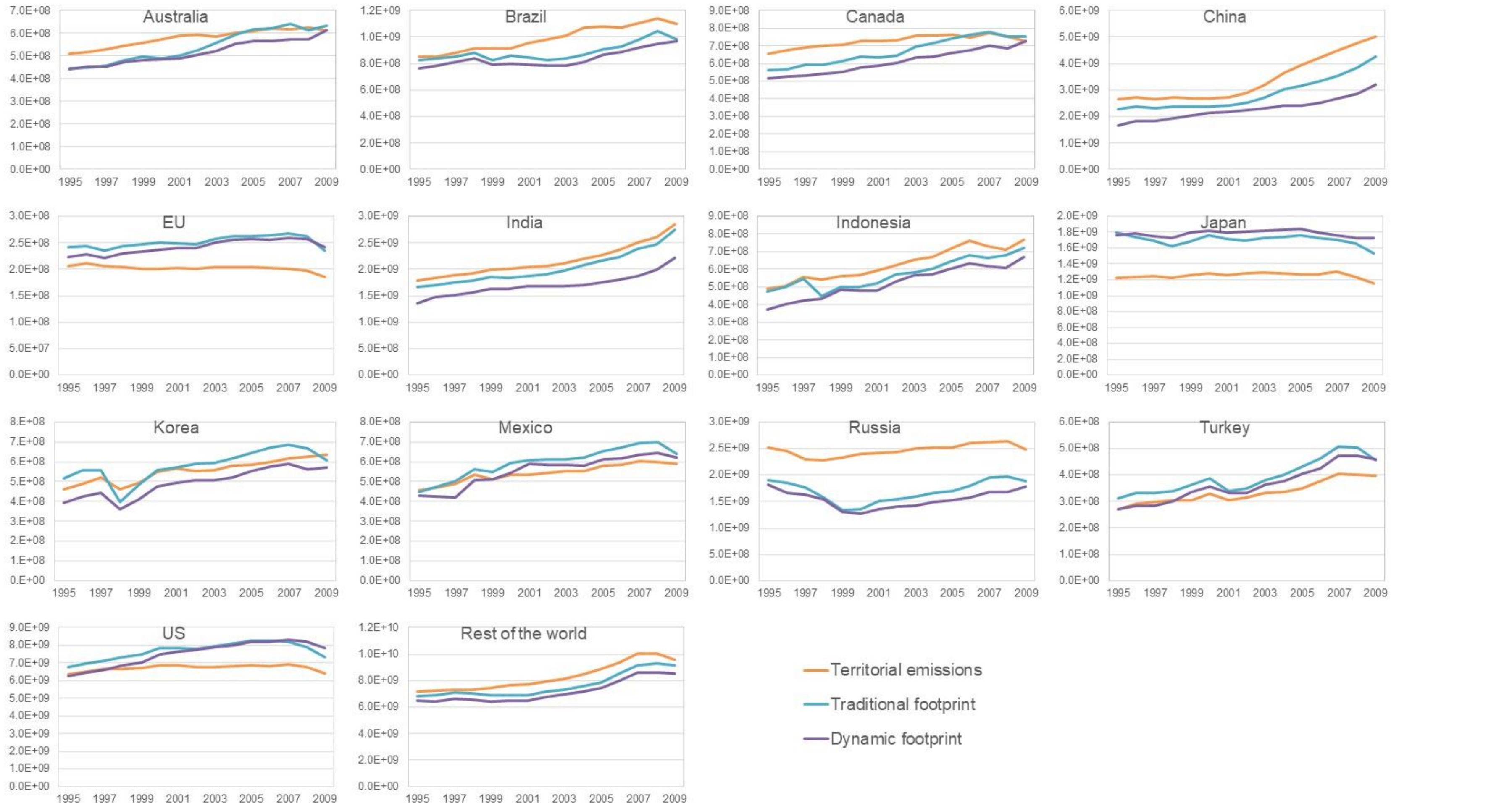

Supplementary Figure 2 Comparison of territorial emissions, traditional footprint, and consumptive footprint. Unit of vertical axis is tCO_2e.

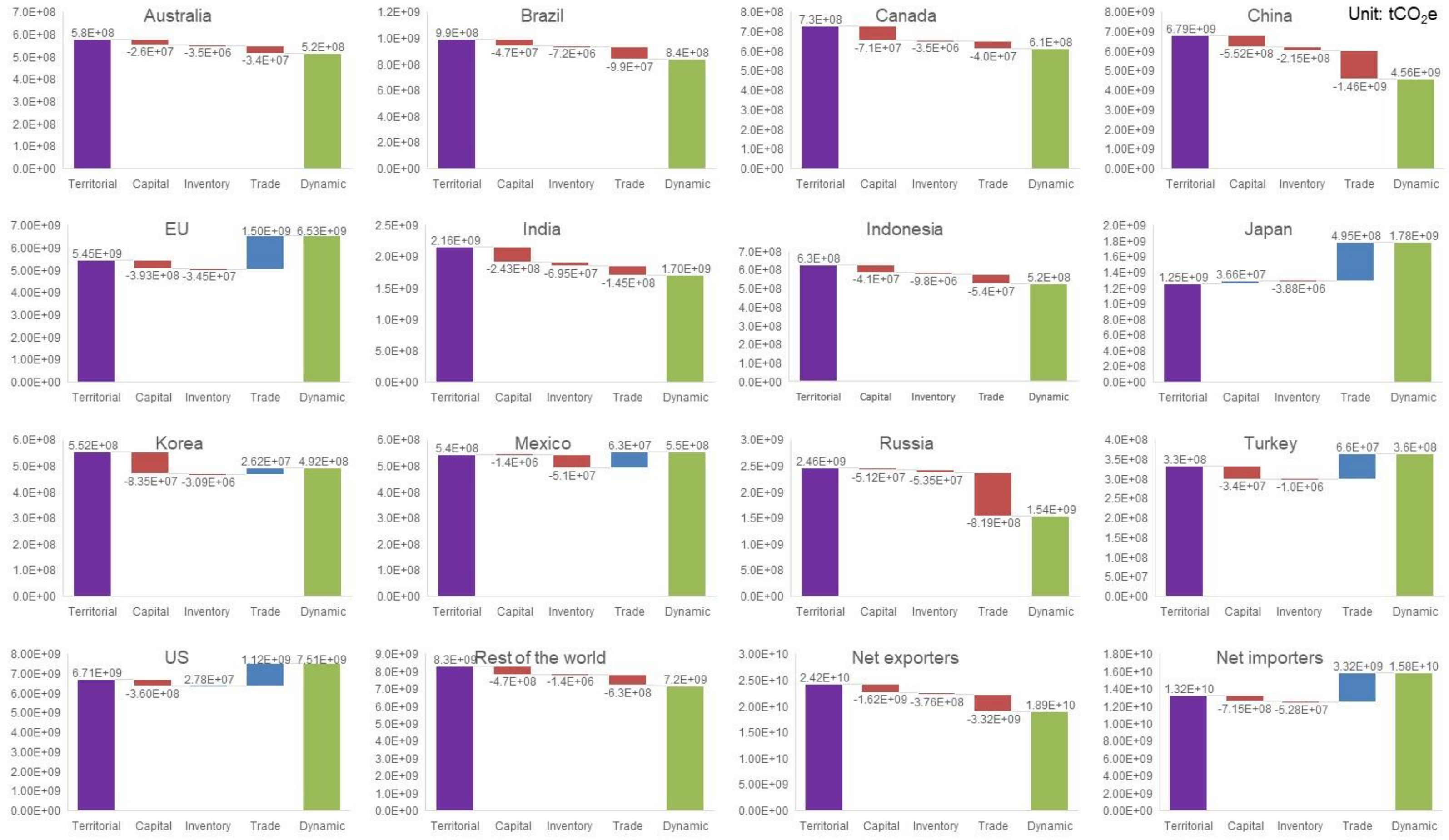

Supplementary Figure 3 Decomposition of the deviation between territorial emissions and consumptive footprint (annual average 1995-2009). Territorial indicates territorial emissions; capital indicates emissions embodied in capital stock change; inventory indicates emissions embodied in inventory change; trade indicates emissions embodied in trade balance; and dynamic indicates dynamic footprint.

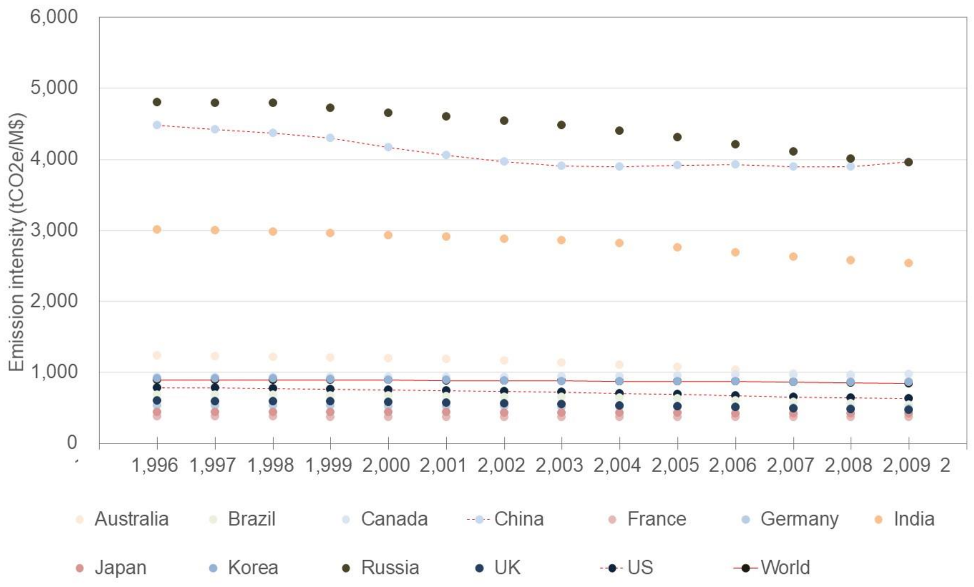

Supplementary Figure 4 Evolution of emission intensity of capital stock of selected economies. Monetary values are in constant 1995 USD.

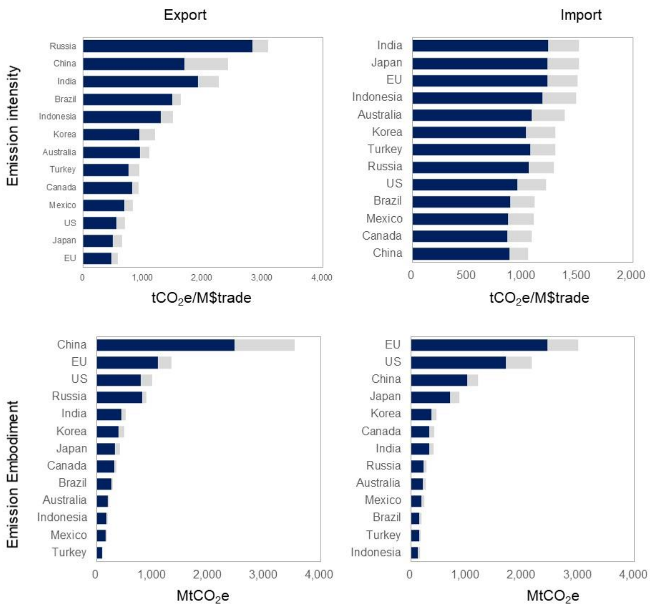

Supplementary Figure 5 Emission intensity and embodiment of trade in 2009. Monetary value is in current USD. The length of the bar indicates totals, the portion in grey indicates cumulative emissions (intensities) before 2009 and that in dark blue indicates current emissions (intensities) in 2009.

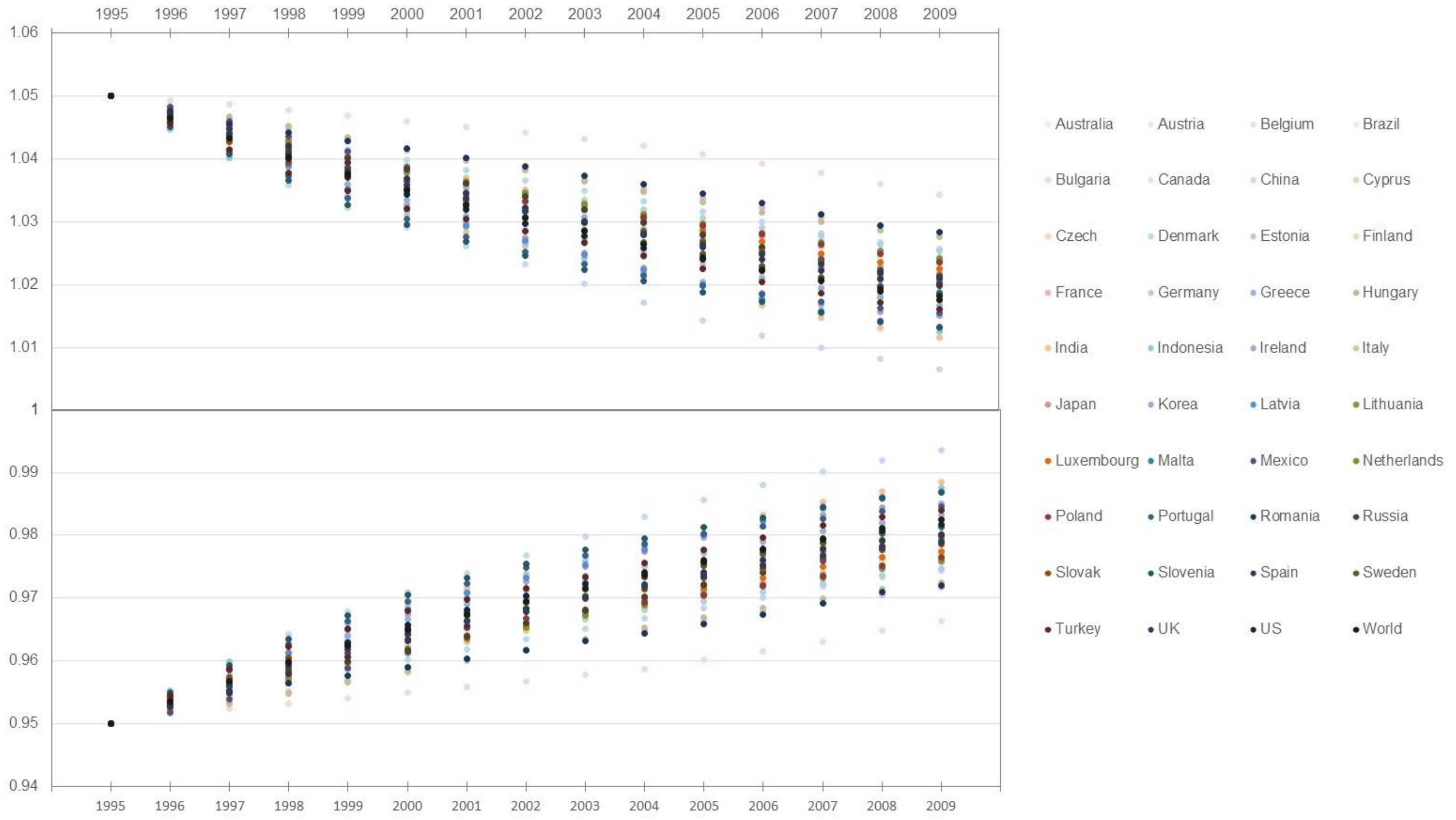

Supplementary Figure 6 Comparison of emission intensity of capital stock based on different initial conditions. Vertical axis indicates ratio of intensity based on the original assumptions to that based on the new assumptions. The upper and lower parts present results based on initial conditions (S1) and (S2), respectively.

Supplementary Tables

Supplementary Table 1 Mapping between the 35 sectors in the original database and the 8 aggregated sectors.

Sector code	Sector name	Aggregated sector name
1	Agriculture, hunting, forestry and fishing	Agriculture
2	Mining and quarrying	Heavy industries
3	Food, beverages and tobacco	Light industries
4	Textiles and textile products	Light industries
5	Leather, leather products and footwear	Light industries
6	Wood and products of wood and cork	Light industries
7	Pulp, paper, printing and publishing	Light industries
8	Coke, refined petroleum and nuclear fuel	Heavy industries
9	Chemicals and chemical products	Heavy industries
10	Rubber and plastics	Heavy industries
11	Other non-metallic mineral	Heavy industries
12	Basic metals and fabricated metal	Heavy industries
13	Machinery, not elsewhere classified	Machinery
14	Electrical and optical equipment	Machinery
15	Transport equipment	Machinery
16	Manufacturing, not elsewhere classified; recycling	Machinery
17	Electricity, gas and water supply	Utilities
18	Construction	Construction
19	Sale and repair of motor vehicles and motorcycles; retail sale of fuel	Business services
20	Wholesale trade, except of motor vehicles and motorcycles	Business services
21	Retail trade and repair, except of motor vehicles and motorcycles;	Business services
22	Hotels and restaurants	Business services
23	Inland transport	Business services
24	Water transport	Business services
25	Air transport	Business services
26	Other supporting transport activities	Business services
27	Post and telecommunications	Business services
28	Financial intermediation	Business services
29	Real estate activities	Business services
30	Renting of machinery & equipment and other business activities	Business services
31	Public administration and defence; compulsory social security	Social services
32	Education	Social services
33	Health and social work	Social services
34	Other community, social and personal services	Social services
35	Private households with employed persons	Business services

Supplementary Table 2 Decomposition of the change of global emissions embodied in capital stock.

	Capital stock expansion	International capital share change	Emission intensity change
1995-1996	1,897	646	0
1996-1997	2,044	526	-181
1997-1998	2,074	338	-109
1998-1999	2,216	277	-358
1999-2000	2,321	191	-653
2000-2001	2,093	198	-625
2001-2002	1,951	215	-653
2002-2003	2,075	406	-742
2003-2004	2,491	634	-861
2004-2005	2,985	792	-1,118
2005-2006	3,516	917	-1,504
2006-2007	4,071	1,204	-2,353
2008-2008	4,217	1,597	-2,892
2008-2009	2,889	1,423	-1,865
Sum of above	36,838	9,364	-13,914

Note: Emission intensity change does not contribute to the change between 1995 and 1996 because of the initial condition assumption of constant intensity (see Methods for details).

Supplementary Table 3 Data availability and aggregation information for the 41 regions.

Region	Capital 1995-2007	Capital 2008	Capital 2009	Aggregation
Australia	Yes	Yes	Yes	
Austria	Yes			EU
Belgium	Yes	Yes	Yes	EU
Brazil	Yes	Yes	Yes	
Bulgaria	Yes	Yes	Yes	EU
Canada	Yes	Yes	Yes	
Cyprus	Yes	Yes		EU
Czech	Yes			EU
Denmark	Yes			EU
Estonia	Yes	Yes	Yes	EU
Finland	Yes			EU
France	Yes			EU
Germany	Yes			EU
Greece	Yes	Yes	Yes	EU
Hungary	Yes			EU
India	Yes	Yes	Yes	
Indonesia	Yes	Yes	Yes	
Ireland	Yes			EU
Italy	Yes			EU
Japan	Yes	Yes	Yes	
Korea	Yes	Yes	Yes	
Latvia	Yes	Yes	Yes	EU
Lithuania	Yes	Yes	Yes	EU
Luxembourg	Yes	Yes	Yes	EU
Mainland, China	Yes	Yes	Yes	China
Malta	Yes	Yes	Yes	EU
Mexico	Yes	Yes	Yes	
Netherlands	Yes			EU
Poland	Yes			EU
Portugal	Yes	Yes		EU
Romania	Yes	Yes	Yes	EU
Russia	Yes	Yes	Yes	
Slovak	Yes	Yes	Yes	EU
Slovenia	Yes	Yes	Yes	EU
Spain	Yes			EU
Sweden	Yes			EU
Taiwan, China	Yes	Yes	Yes	China
Turkey	Yes	Yes	Yes	
UK	Yes			EU
US	Yes	Yes	Yes	
Rest of the world				

Note: Yes for the second to the fourth column indicates sectoral capital stock data are available for the periods 1995-2007, 2008, and 2009, respective. EU and China in the fifth column indicates the regions are aggregated to the EU and China, respectively in our analyses.

Supplementary Table 4 Emission intensity of capital stock (unit: $tCO_2e/M\$$).

Region	1996	1997	1998	1999	2000	2001	2002	2003	2004	2005	2006	2007	2008	2009
Australia	1,232	1,227	1,219	1,206	1,194	1,180	1,160	1,133	1,104	1,073	1,035	984	932	886
Austria	558	556	554	551	547	543	538	535	532	529	524	518	513	508
Belgium	564	562	561	560	559	557	554	554	555	555	555	555	557	555
Brazil	682	680	678	672	666	658	653	647	638	626	605	579	555	536
Bulgaria	4,981	4,951	4,896	4,826	4,724	4,594	4,460	4,321	4,160	3,986	3,796	3,614	3,430	3,358
Canada	932	931	933	935	935	937	937	941	947	954	961	966	968	971
China	4,476	4,423	4,373	4,295	4,170	4,059	3,965	3,903	3,898	3,920	3,930	3,895	3,892	3,961
Cyprus	1,162	1,168	1,173	1,196	1,216	1,222	1,218	1,222	1,233	1,237	1,236	1,231	1,251	1,242
Czech	1,668	1,663	1,637	1,606	1,567	1,521	1,482	1,449	1,411	1,374	1,331	1,290	1,254	1,221
Denmark	469	473	469	463	456	449	440	433	426	416	404	392	380	370
Estonia	2,077	2,047	1,999	1,982	1,957	1,908	1,887	1,856	1,806	1,748	1,671	1,611	1,559	1,531
Finland	684	679	673	670	665	661	657	656	654	650	646	641	636	631
France	377	375	374	373	372	370	368	367	366	365	365	365	364	363
Germany	532	530	528	525	523	519	515	512	510	507	502	497	492	486
Greece	1,012	1,007	1,001	990	981	967	959	948	937	925	911	898	891	883
Hungary	1,455	1,448	1,432	1,420	1,399	1,373	1,348	1,322	1,291	1,256	1,218	1,179	1,138	1,100
India	3,011	3,003	2,981	2,959	2,930	2,911	2,882	2,856	2,813	2,758	2,684	2,624	2,572	2,534
Indonesia	1,354	1,346	1,348	1,390	1,397	1,419	1,447	1,485	1,517	1,535	1,574	1,604	1,625	1,663
Ireland	661	655	643	627	604	582	559	541	527	509	495	484	474	465
Italy	580	579	579	578	576	573	570	569	569	567	565	563	561	557
Japan	444	442	439	439	439	438	434	431	429	426	423	421	418	418
Korea	917	916	909	903	896	889	881	877	874	871	868	865	862	857
Latvia	1,271	1,243	1,263	1,304	1,326	1,337	1,331	1,320	1,311	1,297	1,289	1,305	1,321	1,328

Lithuania	2,130	2,117	2,091	2,088	2,068	2,038	2,006	1,972	1,911	1,843	1,775	1,709	1,652	1,623
Luxembourg	523	520	517	515	508	500	492	488	488	488	487	486	478	471
Malta	696	688	684	684	682	687	689	696	700	705	717	724	731	742
Mexico	1,097	1,100	1,111	1,122	1,138	1,157	1,172	1,180	1,187	1,196	1,206	1,212	1,215	1,218
Netherlands	643	639	635	630	624	615	608	602	596	589	580	567	557	547
Poland	1,945	1,924	1,888	1,852	1,812	1,775	1,735	1,697	1,654	1,614	1,571	1,527	1,487	1,441
Portugal	761	757	751	741	730	720	712	703	695	687	675	661	649	638
Romania	4,519	4,462	4,378	4,296	4,211	4,120	4,028	3,933	3,810	3,685	3,535	3,361	3,175	3,049
Russia	4,801	4,797	4,796	4,727	4,654	4,600	4,542	4,479	4,397	4,305	4,209	4,107	4,011	3,961
Slovak	2,130	2,098	2,068	2,044	2,013	1,973	1,941	1,920	1,893	1,867	1,831	1,795	1,757	1,726
Slovenia	798	793	790	784	778	769	762	757	747	738	727	716	713	707
Spain	580	579	577	576	574	572	571	571	572	574	574	574	571	570
Sweden	475	472	469	466	463	459	456	457	455	452	449	445	441	436
Turkey	1,184	1,180	1,185	1,196	1,205	1,215	1,221	1,225	1,225	1,217	1,212	1,205	1,210	1,203
UK	596	595	593	589	581	570	558	547	534	521	507	492	478	466
US	784	781	776	765	752	741	730	718	704	690	675	655	638	625
World	889	892	894	894	889	885	881	878	876	874	869	860	850	847

Note: Monetary values are in constant 1995 USD.

Supplementary Table 5 Total emissions embodied in bilateral trade flows in 2009 (unit: $MtCO_2e$).

Exporter\Importer	Australia	Brazil	Canada	China	EU	India	Indonesia	Japan	Korea	Mexico	Russia	Turkey	US
Australia	-	0.68	5.27	56.74	21.04	9.62	7.44	30.69	14.87	0.48	0.73	0.14	14.98
Brazil	0.85	-	5.37	55.66	74.21	3.02	1.99	8.21	5.69	2.46	5.71	1.75	22.04
Canada	3.33	4.86	-	21.39	46.65	4.54	1.26	12.46	3.92	7.53	0.88	0.63	199.74
China	108.59	44.88	108.15	-	777.60	122.15	55.21	354.72	178.38	70.13	70.11	38.44	749.06
EU	26.65	25.47	37.24	108.13	-	19.61	7.22	37.09	25.99	18.95	61.68	40.98	194.77
India	10.72	4.24	30.78	35.27	138.17	-	8.77	17.65	5.95	2.92	5.86	9.41	110.88
Indonesia	6.16	1.25	1.75	26.71	33.61	7.25	-	26.32	11.63	0.80	1.39	3.59	16.32
Japan	6.48	2.60	6.38	95.02	44.53	2.50	6.85	-	33.28	5.44	4.52	0.88	46.15
Korea	6.49	4.53	6.13	128.34	62.53	5.99	6.82	30.17	-	9.01	6.89	4.13	43.48
Mexico	0.58	1.80	10.27	3.63	14.66	0.34	0.09	2.00	0.63	-	0.20	0.18	130.42
Russia	0.86	3.82	2.63	65.94	396.02	5.13	1.20	22.42	14.15	1.16	-	12.46	33.63
Turkey	0.43	0.42	0.86	1.68	55.78	0.71	0.30	0.42	0.34	0.21	4.14	-	4.53
US	14.53	16.68	137.16	98.40	206.67	14.02	5.22	61.39	25.97	90.88	5.39	4.56	-

Supplementary Table 6 Cumulative emissions embodied in bilateral trade flows in 2009 (unit: $MtCO_2e$).

Exporter\Importer	Australia	Brazil	Canada	China	EU	India	Indonesia	Japan	Korea	Mexico	Russia	Turkey	US
Australia	-	0.11	0.91	8.10	3.62	1.33	0.72	4.16	2.24	0.09	0.11	0.02	2.33
Brazil	0.10	-	0.56	3.82	5.84	0.23	0.12	0.65	0.43	0.42	0.28	0.12	2.84
Canada	0.38	0.56	-	2.41	5.69	0.47	0.14	1.25	0.44	1.00	0.14	0.07	25.52
China	32.89	13.46	31.96	-	246.84	39.18	16.30	98.85	52.99	21.52	21.94	11.30	232.62
EU	4.36	5.15	6.95	23.25	-	3.87	1.34	6.88	5.42	3.42	11.68	7.34	37.17
India	1.62	0.65	2.87	4.52	22.00	-	1.43	2.14	0.79	0.49	0.93	1.59	21.32
Indonesia	0.84	0.19	0.28	2.70	5.84	0.70	-	2.82	0.95	0.14	0.24	0.56	2.53
Japan	1.75	0.72	1.88	25.76	12.57	0.63	1.64	-	7.12	1.49	1.47	0.25	13.24
Korea	1.35	1.11	1.47	31.37	16.40	1.35	1.35	6.00	-	2.45	1.67	0.92	10.52
Mexico	0.11	0.32	1.96	0.71	2.49	0.05	0.02	0.27	0.10	-	0.04	0.03	22.61
Russia	0.08	0.29	0.17	5.34	31.79	0.41	0.09	1.82	1.15	0.08	-	0.99	2.62
Turkey	0.07	0.08	0.15	0.35	11.93	0.12	0.04	0.07	0.07	0.05	0.94	-	0.80
US	3.17	3.10	27.77	17.91	46.94	3.59	0.81	9.16	5.24	16.07	1.04	0.55	-

Supplementary Methods

While the initial condition leading to Equation (5) in the Methods section is determined based on the seemingly reasonable but arbitrary constant intensity assumptions, this section provides additional information regarding how the emission intensity of capital stock will be affected when the initial conditions are changed. The annual change of emission intensity of capital stock for the 39 countries from the original model varies between -5.6% and 3.2%. Only two annual changes exceed 5%: Bulgaria (-5.1%) and Romania (-5.6%), both from 2007 to 2008. Therefore, we examine how the results will be affected by setting initial conditions of:

$$\begin{cases} \bar{\varepsilon}_{k,t_0+1} = 1.05 \times \bar{\varepsilon}_{k,t_0} \\ \varepsilon_{i,t_0+1} = \varepsilon_{i,t_0} \end{cases} \qquad \text{(S1)}$$

and

$$\begin{cases} \bar{\varepsilon}_{k,t_0+1} = 0.95 \times \bar{\varepsilon}_{k,t_0} \\ \varepsilon_{i,t_0+1} = \varepsilon_{i,t_0} \end{cases} \qquad \text{(S2)}$$

We keep the sectoral emission intensity constant because it can largely simplify the calculation while the effect is tiny because the emissions embodied in inventory input are very small comparing with those in capital depreciation. Our calculation shows that the emission intensity of capital stock converges on results based on the original assumptions (Supplementary Figure 6). For the world as a whole, the deviation of the new intensity from the old one becomes smaller than 2% after 14 years (since 2008). To conclude, when no obvious external shock (e.g., war, serious disaster, or serious depression) is imposed on an economy, the initial condition assumptions of constant intensity are deemed to be reasonable.
